# Assessment of Knowledge and Attitudes of Parents Regarding Neonatal Jaundice in Abia State Children’s Specialist Hospital, Umuahia, Nigeria: A Cross-Sectional Study

**DOI:** 10.7759/cureus.69163

**Published:** 2024-09-11

**Authors:** Chimaobi Ezekiel Ijioma, Ngozi Uloma Enwereji, Oladoyin Ogunbayo Jolaoye, Prosper Chisom Okebugwu, Osarumwense D Ufuah, Ifeoma Chinaemerem Ezirim, Cynthia Kenechukwu Madueke, Abasiekeme Monday Ekwere, Excel Nwasinachi Victor-Anozie, Innocent Chima Zacs, Ochuko Austin-Jemifor, Chisom Anthonia Onua, Ifeanyichukwu Williams Uwalaka

**Affiliations:** 1 Pediatrics, Abia State Children's Specialist Hospital, Umuahia, NGA; 2 Internal Medicine - Pediatrics, Order of Saint Francis Medical Center, University of Illinois College of Medicine at Peoria, Peoria, USA; 3 Family Medicine, Hennepin Healthcare, Minneapolis, USA; 4 General Practice, All Saints University College of Medicine, Kingstown, VCT; 5 Family Medicine, Alex Ekwueme Federal University Teaching Hospital, Abakaliki, NGA; 6 Pediatrics, American University of Barbados, Wildey, BRB; 7 Family Medicine, All Saints University, Roseau, DMA; 8 General Medicine, St. Joseph Catholic Hospital, Asaba, NGA; 9 Internal Medicine, Abia State University Teaching Hospital, Aba, NGA; 10 Internal Medicine, Queen Elizabeth Hospital Birmingham, University Hospitals Birmingham, National Health Service Foundation Trust, Birmingham, GBR; 11 Pediatrics, Sangotedo Primary Health Care Center, Sangotedo, NGA; 12 Internal Medicine, Diamed Centre, Lekki, NGA

**Keywords:** attitudes, health education, neonatal jaundice, parental knowledge, pediatric health

## Abstract

Background

Neonatal jaundice, characterized by the yellow discoloration of an infant’s skin and eyes, is a common condition that affects newborns. It results from an elevated level of bilirubin in the blood and, when severe, if left untreated, can lead to complications such as acute bilirubin encephalopathy and kernicterus, which can cause permanent neurological damage or even death. In low-resource settings like Nigeria, delayed recognition and inadequate management of neonatal jaundice are significant contributors to neonatal morbidity and mortality. Parental knowledge and attitudes play a critical role in the early identification and timely intervention of neonatal jaundice. However, there is limited data on the awareness, understanding, and practices of parents regarding this condition in many regions, including Abia State, Nigeria.

Aim

This study sought to assess the knowledge and attitudes of parents at Abia State Children's Specialist Hospital, Umuahia, Nigeria, to inform strategies for improving early detection and management of neonatal jaundice.

Research methodology

This descriptive cross-sectional study was conducted at Abia State Children's Specialist Hospital. The study population included 385 parents (fathers and mothers) of neonates attending the pediatric and neonatal units. A systematic random sampling technique was used to select participants. Data were collected using a structured questionnaire and analyzed using IBM SPSS Statistics for Windows, Version 25, (Released 2017; IBM Corp., Armonk, New York, United States).

Results

The study revealed that 226 (58.70%) of parents had heard about neonatal jaundice, with the main perceived cause being an immature liver (148, 38.44%). While 216 (56.10%) of the parents recognized neonatal jaundice as a serious condition when severe, only 72 (18.70%) were very confident in identifying its signs. Most parents (369, 95.84%) would seek medical help if they suspected jaundice in their baby, yet 288 (74.81%) believed in the effectiveness of traditional remedies. Educational level and gender were significantly associated with knowledge about neonatal jaundice (p = 0.002 and p = 0.001, respectively).

Conclusion

The findings highlight a moderate level of awareness but varying confidence in identifying neonatal jaundice among parents. The reliance on traditional remedies suggests a need for enhanced health education to improve knowledge of and attitudes toward neonatal jaundice management.

## Introduction

Neonatal jaundice, a common condition in newborns, is characterized by the yellowing of the skin and eyes due to elevated bilirubin levels. While often benign, severe cases can lead to serious complications, such as acute bilirubin encephalopathy and kernicterus, which can result in permanent neurological damage or death [1]. Early recognition and management are crucial to preventing these adverse outcomes. Despite advancements in healthcare, neonatal jaundice remains a significant public health concern, especially in low- and middle-income countries, including Nigeria [2].

In Nigeria, neonatal jaundice accounts for a substantial proportion of neonatal morbidity and mortality [3]. Several factors, including the level of parental knowledge and attitudes toward the condition, influence the prevalence and outcomes of neonatal jaundice. Parents' awareness and understanding of neonatal jaundice are critical, as they are often the first to notice symptoms and seek medical intervention [4]. However, studies have shown that many parents in developing countries lack adequate knowledge about neonatal jaundice, its causes, and the importance of timely medical care [2].

Abia State Children's Specialist Hospital, Umuahia, Nigeria, serves as a referral center for many neonatal conditions, including jaundice. Understanding the knowledge and attitudes of parents regarding neonatal jaundice in this setting is essential for developing targeted educational interventions to improve health outcomes. Previous studies conducted in different parts of Nigeria have highlighted significant gaps in parents' knowledge about neonatal jaundice [2,3]. However, there is limited research focusing specifically on Abia State, Nigeria, necessitating a localized assessment to inform healthcare policies and practices in this region.

Parents' knowledge of and attitudes toward neonatal jaundice are shaped by various factors, including educational background, socio-economic status, cultural beliefs, and previous experiences with the condition [5]. Misconceptions and cultural myths can hinder the effective management of neonatal jaundice, leading to delays in seeking appropriate care and poor adherence to treatment [6]. Additionally, the role of healthcare professionals in educating parents and dispelling myths cannot be overemphasized. Studies have shown that parents who receive adequate information from healthcare providers are more likely to recognize jaundice early and adhere to recommended treatment protocols [5].

The aim of this study was to assess the knowledge and attitudes of parents regarding neonatal jaundice at Abia State Children's Specialist Hospital. By identifying gaps in knowledge and misconceptions, the study aims to provide insights into the need for the development of effective educational programs and strategies to improve parental awareness and engagement in the management of neonatal jaundice. Such initiatives are expected to enhance early detection and prompt treatment, ultimately reducing the burden of neonatal jaundice in this region.

## Materials and methods

Study design

This study employed a descriptive cross-sectional design to assess the knowledge and attitudes of parents regarding neonatal jaundice in Abia State Children's Specialist Hospital.

Study setting

This cross-sectional study was conducted at the Abia State Children's Specialist Hospital from November 23, 2023, to June 14, 2024. This hospital is a major pediatric healthcare center serving Umuahia and its surrounding areas.

Study participants and eligibility criteria

The study participants included parents (both mothers and fathers) of neonates attending the pediatric and neonatal units of Abia State Children's Specialist Hospital.

Inclusion criteria

Participants were parents (fathers and mothers) of neonates aged 0-28 days who were admitted to or seen at the facility for general medical conditions and who provided informed consent to participate in the study.

Exclusion criteria

Participants excluded from the study were parents of neonates with severe medical conditions requiring immediate intensive care and those who refused to provide consent.

Sample size determination

The sample size for this study was calculated using the Cochran formula for estimating proportions in a population, as outlined by Airaodion et al. [7]. The formula used was n = (Z² * P * q) / e², where n represented the minimum sample size, Z was the Z-score at a 95% confidence level (1.96), P was the known prevalence of neonatal jaundice in Nigeria, e was the margin of error tolerated at 5% (0.05), and q was equal to 1 - P. According to Onyearugha et al., the prevalence of neonatal jaundice in southeast Nigeria is 35% (P = 0.35) [8], making q equal to 0.65. Substituting these values into the formula, the calculation was n = (1.96² * 0.35 * 0.65) / (0.05)², resulting in a minimum sample size of 349.59, which was rounded up to 350. To account for a potential non-response rate of 10%, the sample size was further adjusted to 385.

Sampling technique

A systematic random sampling technique was employed. The sampling frame was the list of neonates registered at the neonatal units during the study period. One out of every two parents who met the inclusion criteria was selected until the sample size was achieved.

Data collection instrument

A structured questionnaire was used to collect data. The questionnaire was developed based on literature reviews and expert consultations. The questionnaire was pre-tested on a sample of 30 parents from a different hospital to assess its validity and reliability. Cronbach's alpha was used to measure the internal consistency of the knowledge and attitude sections, with a value of 0.7. The questionnaire covered the demographic information of the participants, knowledge about neonatal jaundice, attitudes toward neonatal jaundice, and information sources and education.

Data analysis

Data were analyzed using IBM SPSS Statistics for Windows, Version 25, (Released 2017; IBM Corp., Armonk, New York, United States). Descriptive statistics (frequencies and percentages) were used to summarize the demographic characteristics, knowledge, and attitudes of parents. A chi-square test was employed to assess associations between demographic variables and knowledge. A p-value of <0.05 was considered statistically significant.

Ethical approval and consent to participate

Ethical approval was obtained from the Ethics Committee of Abia State Children's Specialist Hospital, Umuahia, Nigeria, with approval number ASCSH/EC/23/029 on October 29, 2023. Written informed consent was obtained from all participants. Participants' confidentiality was maintained by using codes instead of names and storing data securely. Participation was voluntary, and participants could withdraw without any consequences.

## Results

The sample included 385 participants. The majority were 26-35 years old (248, 64.42%), followed by those 36-45 years old (99, 25.71%). Most participants were female (306, 79.48%) and married (347, 90.13%). Education levels varied, with 219 (56.88%) having secondary education and 89 (23.12%) having tertiary education. The majority were self-employed (212, 55.06%), as shown in Table 1.

**Table 1:** Demographic information of the participants

Variable	Frequency (n = 385)	Percentage (%)
Age		
18-25 years	36	9.35
26-35 years	248	64.42
36-45 years	99	25.71
46 years and above	2	0.52
Gender		
Male	79	20.52
Female	306	79.48
Marital status		
Single	27	7.01
Married	347	90.13
Divorced/widowed	11	2.86
Education level		
No formal education	25	6.49
Primary education	52	13.51
Secondary education	219	56.88
Tertiary education	89	23.12
Occupation		
Unemployed	11	2.86
Self-employed	212	55.06
Private sector employee	74	19.22
Public sector employee	86	22.34
Student	2	0.52

**Figure 1:**
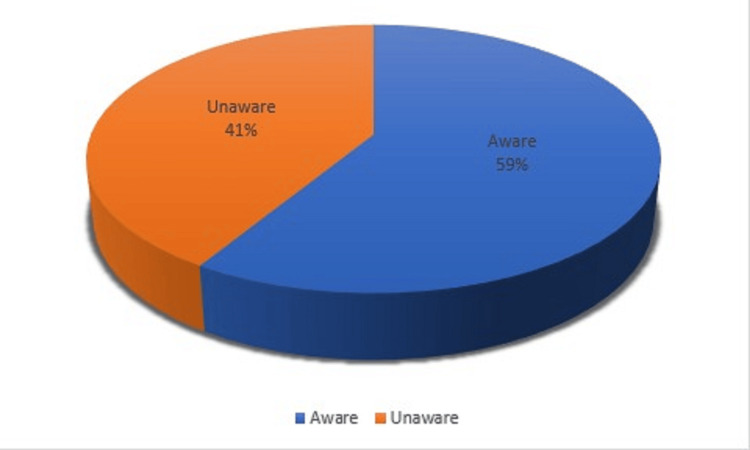
Awareness of neonatal jaundice Data are presented in %

The findings presented in Figure 1 revealed that 226 (58.70%) of the participants were aware of what neonatal jaundice is, whereas 159 (41.3%) were not aware of what it is.

Most participants commonly identified the cause of neonatal jaundice as an immature liver (148, 38.44%). Yellowing of the skin and eyes was the most recognized symptom (296, 42.35%). Premature babies were identified as the highest-risk group (219, 37.24%), and brain damage was the most frequently acknowledged complication of untreated neonatal jaundice (202, 26.47%). Diagnostic methods varied, with blood tests being the most recognized (311, 27.64%). For treatment, medication was most commonly known (346, 33.01%). Monitoring bilirubin levels was seen as the most effective prevention method (321, 42.91%), as shown in Table 2.

**Table 2:** Knowledge about neonatal jaundice * indicates multiple responses

Variable	Frequency	Percentage (%)
What is the main cause of neonatal jaundice?		
Infection	109	28.31
Immature liver	148	38.44
Poor hygiene	101	26.23
Others (please specify)	27	7.01
*What are the common signs and symptoms of neonatal jaundice? (Select all that apply) (n = 699)		
Yellowing of the skin and eyes	296	42.35
Poor feeding	114	16.31
High-pitched crying	85	12.16
Lethargy	78	11.16
Fever	126	18.03
*Which newborns are at higher risk for neonatal jaundice? (Select all that apply) (n = 588)		
Premature babies	219	37.24
Babies with a different blood type from the mother	23	3.91
Babies with siblings who had jaundice	185	31.46
Babies who are not feeding well	161	27.38
*What are the potential complications of untreated neonatal jaundice? (Select all that apply) (n = 763)		
Brain damage	202	26.47
Hearing loss	186	24.38
Cerebral palsy	148	19.40
Developmental delay	227	29.75
*How is neonatal jaundice diagnosed? (Select all that apply) (n = 1125)		
Physical examination	210	18.67
Blood test	311	27.64
Urine test	298	26.49
Stool test	306	27.20
*What treatments are available for neonatal jaundice? (Select all that apply) (n = 1048)		
Phototherapy (light treatment)	303	28.91
Exchange transfusion	184	17.56
Medication	346	33.01
Increased feeding as an enhancer	164	15.65
None of the above	51	4.87
*How can neonatal jaundice be prevented? (Select all that apply) (n = 748)		
Early and frequent breastfeeding	125	16.71
Monitoring bilirubin levels	321	42.91
Immunization	114	15.24
Maintaining hygiene	188	25.14

A slight majority (216, 56.10%) viewed neonatal jaundice as a serious condition when severe, and 369 (95.84%) would seek medical help if they suspected jaundice in their baby. A significant portion (288, 74.81%) believed in the effectiveness of traditional remedies, yet all participants indicated they would follow healthcare provider advice. Education on neonatal jaundice was deemed very important by 304 (78.96%) of the respondents, as shown in Table 3.

**Table 3:** Attitudes toward neonatal jaundice

Variable	Frequency (n = 385)	Percentage (%)
Do you think neonatal jaundice when severe is a serious condition?		
Yes	216	56.10
No	71	18.44
Unsure	98	25.45
How confident are you in your ability to identify signs of neonatal jaundice in a newborn?		
Very confident	72	18.70
Confident	103	26.75
Neutral	106	27.53
Not confident	58	15.06
Not confident at all	46	11.95
Would you seek medical help if you suspected your baby had jaundice?		
Yes	369	95.84
No	0	0.00
Unsure	16	4.16
Where would you first seek help if you noticed signs of jaundice in your baby?		
Hospital/clinic	225	58.44
Traditional healer	13	3.38
Pharmacy	85	22.08
Family/friends	40	10.39
Others	22	5.71
Do you believe traditional remedies are effective for treating neonatal jaundice?		
Yes	288	74.81
No	57	14.81
Unsure	40	10.39
Would you follow a healthcare provider's advice if your baby was diagnosed with jaundice?		
Yes	385	100.00
No	0	0.00
Unsure	0	0.00
How important do you think it is for parents to be educated about neonatal jaundice?		
Very important	304	78.96
Important	72	18.70
Neutral	09	2.34
Not important	00	0.00
Not important at all	00	0.00

Most participants (303, 78.70%) had received information about neonatal jaundice, primarily from healthcare providers (289, 54.84%). All participants expressed a desire for more information, preferring to receive it via the Internet or social media (324, 45.06%). A significant majority (337, 87.53%) had attended health education programs, with 298 (88.43%) of these programs including information on neonatal jaundice, as shown in Table 4.

**Table 4:** Information sources and education * indicates multiple responses

Variable	Frequency	Percentage (%)
Have you ever received information about neonatal jaundice?		
Yes	303	78.70
No	82	21.30
*If yes, where did you receive this information? (Select all that apply) (n = 527)		
Healthcare providers	289	54.84
Family and friends	54	10.25
Internet	112	21.25
Books/magazines	16	3.04
Community health programs	56	10.63
Would you like to receive more information about neonatal jaundice?		
Yes	385	100.00
No	0	0.00
*What is your preferred method for receiving information about neonatal jaundice? (Select all that apply) (n = 719)		
Healthcare providers	183	25.45
Community health programs	42	5.84
Internet/social media	324	45.06
Printed materials (brochures, leaflets)	42	5.84
Television/radio	128	17.80
Have you ever attended any health education programs regarding newborn care?		
Yes	337	87.53
No	48	12.47
If yes, did the program include information about neonatal jaundice?		
Yes	298	88.43
No	39	11.57

Knowledge of neonatal jaundice was significantly associated with age, gender, and education level. Younger participants (18-25 years) and males were less likely to have heard of neonatal jaundice compared to older participants and females. Those with tertiary education were more likely to be knowledgeable compared to those with lower educational levels. Occupation and marital status were not significantly associated with knowledge levels, as shown in Table 5.

**Table 5:** Factors affecting the knowledge of neonatal jaundice in Abia State, Nigeria p<0.05 is statistically significant

Variable	Have you heard about neonatal jaundice?		p-value
	Yes (n = 226)	No (n = 159)	
Age			0.001
18-25 years	6 (16.67%)	30 (83.33%)	
26-35 years	153 (61.69%)	95 (38.31%)	
36-45 years	65 (65.66%)	34 (34.34%)	
46 years and above	2 (100.00%)	0 (0.00%)	
Gender			0.001
Male	17 (21.52%)	62 (78.48%)	
Female	209 (68.30%)	97 (31.70%)	
Marital status			0.165
Single	16 (59.26%)	11 (40.74%)	
Married	204 (58.79%)	143 (41.21%)	
Divorced/widowed	6 (54.55%)	5 (45.45%)	
Education level			0.002
No formal education	4 (16.00%)	21 (84.00%)	
Primary education	10 (19.23%)	42 (80.77%)	
Secondary education	145 (66.21%)	74 (33.79%)	
Tertiary education	67 (75.28%)	22 (24.72%)	
Occupation			0.633
Unemployed	6 (54.55%)	5 (45.45%)	
Self-employed	125 (58.96%)	87 (41.04%)	
Private sector employee	45 (60.81%)	29 (39.19%)	
Public sector employee	49 (56.98%)	37 (43.02%)	
Student	1 (50.00%)	1 (50.00%)	

## Discussion

The results of our study underscore critical insights into the knowledge and attitudes of parents regarding neonatal jaundice at Abia State Children's Specialist Hospital. 

Our study provided valuable insights into the demographic profile of parents attending Abia State Children's Specialist Hospital, and how these characteristics might influence their knowledge of and attitudes toward neonatal jaundice. The majority of participants were aged 26-35 years (64.42%), suggesting a predominantly young parent group. This could affect the generalizability of our findings, as younger parents might have different experiences and levels of awareness compared to older age groups, who were less represented (0.52% aged 46 years and above). Gender differences were notable, with a significant majority of female participants (79.48%) compared to males (20.52%). This disparity likely reflects traditional caregiving roles, where females are more involved in health management and, consequently, may have greater awareness about neonatal conditions. The marital status distribution showed that most respondents were married (90.13%), with fewer being single (7.01%) or divorced/widowed (2.86%). This suggests that the majority of the sample was part of established family units, which could influence their approach to neonatal healthcare.

Educational attainment among participants was relatively high, with a substantial number having secondary (56.88%) or tertiary education (23.12%). The lower proportion of individuals with no formal education (6.49%) highlighted that the majority had at least some level of formal education, which was likely to have contributed to their awareness and understanding of health issues. Regarding occupation, the sample was predominantly self-employed (55.06%), followed by public sector (22.34%) and private sector employees (19.22%). The small percentage of unemployed (2.86%) and students (0.52%) suggests that most participants were engaged in the workforce, which may have influenced their access to and interaction with health information. These demographic factors, age, gender, marital status, education level, and occupation, play a crucial role in shaping parental knowledge and attitudes towards neonatal jaundice. Understanding these characteristics helps identify where targeted educational efforts might be needed to improve awareness and management of neonatal jaundice. Specifically, interventions could focus on addressing the needs of younger parents, males, and those with lower educational attainment to bridge knowledge gaps and enhance health outcomes for neonates.

The study found that 58.70% of parents had heard about neonatal jaundice, while 41.30% had not. This level of awareness was relatively low compared to similar studies in other regions. For example, a study conducted in a tertiary hospital in southwest Nigeria reported a higher awareness rate of 77% [9]. The difference in awareness levels might be attributed to the varying levels of education and access to information across different regions.

Our study highlighted several key aspects of parental knowledge regarding neonatal jaundice. When asked about the main cause of neonatal jaundice, 38.44% of parents correctly identified it as an immature liver, while 28.31% attributed it to infection, and 26.23% believed poor hygiene to be the cause. This suggests a reasonably good understanding of the physiological basis of neonatal jaundice, although there remains some confusion regarding its etiology. Regarding the signs and symptoms of neonatal jaundice, the majority of parents identified yellowing of the skin and eyes (42.35%) as a key indicator. This is consistent with findings from other studies, such as one in Bisha City, Saudi Arabia, where 45% of mothers identified jaundice by the yellowing of the sclera [10]. Other symptoms such as poor feeding (16.31%), fever (18.03%), and high-pitched crying (12.16%) were also recognized, though less frequently. This reflects a partial awareness of the clinical presentation of jaundice, highlighting the need for further education on recognizing less obvious symptoms like lethargy and fever. The lack of awareness of these critical signs could delay diagnosis and treatment, leading to severe complications.

In terms of risk factors, premature babies were identified by 37.24% of parents as being at higher risk which is in line with the medical understanding that premature infants have underdeveloped livers and are, therefore, more prone to jaundice [11]. Only 3.91% recognized babies with a different blood type from the mother as a risk factor. Additionally, 31.46% understood that having siblings who had jaundice is a risk factor, and 27.38% noted poor feeding as a contributing factor. These findings indicate a reasonable grasp of some risk factors but reveal gaps in understanding less common ones including a need for better education on rhesus (Rh) incompatibility and its role in neonatal jaundice. Regarding potential complications of untreated neonatal jaundice, parents identified developmental delay (29.75%) and brain damage (26.47%) as major concerns, with fewer recognizing hearing loss (24.38%) and cerebral palsy (19.40%). This suggests a general awareness of the severe consequences of untreated jaundice, although not all potential complications were equally recognized. Diagnosis methods were well-understood, with blood tests (27.64%), urine tests (26.49%), and stool tests (27.20%) being the most commonly identified methods. Physical examination was less frequently mentioned (18.67%), suggesting a need to emphasize the role of clinical evaluation in diagnosing jaundice.

In terms of treatment, phototherapy (28.91%) and medication (33.01%) were the most recognized, followed by exchange transfusion (17.56%) and increased feeding (15.65%), which suggests a need to emphasize the importance of early and frequent feeding in educational programs. The small proportion who reported 'none of the above' (4.87%) reflects a generally good understanding of available treatments. Finally, preventive measures were also well recognized, with monitoring bilirubin levels (42.91%) being the most cited strategy, followed by maintaining hygiene (25.14%) and early breastfeeding (16.71%). This is concerning, as breastfeeding is known to help reduce the risk of jaundice by promoting the excretion of bilirubin through the digestive system [12]. Immunization was less frequently mentioned (15.24%), indicating an area for potential education. While there is a solid foundation of knowledge regarding neonatal jaundice among parents, there are specific areas where understanding could be improved. Targeted educational efforts could address these gaps, particularly in risk factors, potential complications, and the comprehensive understanding of treatment and prevention strategies.

Our study revealed a range of attitudes and levels of confidence among parents regarding neonatal jaundice. A majority of parents (56.10%) view neonatal jaundice as a serious condition, while a notable proportion remains unsure (25.45%) or does not consider it serious (18.44%). This uncertainty mirrors findings from similar studies conducted in other regions of Nigeria, where misconceptions and a lack of awareness about the potential dangers of neonatal jaundice are common [13]. This suggests a variable perception of the condition’s severity, which could impact how parents respond to potential symptoms and seek care.

Confidence in identifying signs of neonatal jaundice varied among parents, with only 18.70% reporting that they were very confident, and 26.75% feeling confident. A significant number of parents were neutral (27.53%) or lacked confidence in their ability to identify jaundice (27.01%). Comparatively, a study in Lagos, Nigeria, found that only 23% of mothers could correctly identify jaundice in their newborns, indicating a widespread issue of inadequate parental knowledge across the country [14]. This variability indicates a need for enhanced educational efforts to increase parental confidence and competence in recognizing this condition early.

Despite the mixed confidence levels, an overwhelming majority of parents (95.84%) would seek medical help if they suspected their baby had jaundice, reflecting a strong inclination toward professional medical intervention. However, a small proportion (4.16%) remained unsure about seeking help, highlighting a potential area for targeted reassurance and guidance. When seeking initial help, most parents would go to a hospital or clinic (58.44%), while fewer would consult traditional healers (3.38%) or seek advice from pharmacies (22.08%) or family and friends (10.39%). The preference for hospitals and clinics underscores trust in formal medical institutions, though the presence of traditional remedy beliefs (74.81%) indicates a significant segment of the population still values or utilizes non-medical approaches. This belief may contribute to delays in seeking proper medical treatment, as noted in other research conducted in Nigeria and Ghana, where cultural practices and reliance on traditional medicine often interfere with timely medical intervention [13,15]. This underscores the need for culturally sensitive educational campaigns that address these beliefs while promoting the benefits of early medical intervention.

All parents (100%) expressed a willingness to follow healthcare providers' advice if their babies were diagnosed with jaundice, demonstrating a strong commitment to adhering to professional medical recommendations. Furthermore, the majority (78.96%) recognized the importance of educating parents about neonatal jaundice, suggesting a clear understanding of the value of informed caregiving and the potential benefits of targeted educational interventions. While there is a general awareness and proactive attitude toward seeking medical care for neonatal jaundice, the variation in confidence and the role of traditional remedies highlight areas where improved education and integration of culturally sensitive practices could enhance overall management and outcomes for neonatal jaundice.

Our study indicated that a significant majority of parents (78.70%) had received information about neonatal jaundice. The primary sources of this information were healthcare providers (54.84%) and the internet (21.25%), with family and friends (10.25%) and community health programs (10.63%) also contributing. This finding aligns with previous studies that identified healthcare providers as the most trusted and common source of health information [12]. The increasing role of the internet as a source of information is notable and reflects broader trends in digital health information-seeking behaviors observed globally [16]. This underscores the crucial role of healthcare providers in disseminating information and highlights the growing importance of digital resources in patient education.

Despite the substantial percentage of parents who had received information, there was a unanimous interest (100%) in obtaining more information about neonatal jaundice. This suggests a strong demand for further educational resources and underscores the need for ongoing, accessible information. When asked about preferred methods for receiving information, parents showed a clear preference for the Internet and social media (45.06%), reflecting the increasing reliance on digital platforms for health information. Healthcare providers (25.45%) and television/radio (17.80%) were also popular, while community health programs and printed materials were less favored (both 5.84%). The preference for Internet-based information sources reflects global trends in health information seeking. Aldossari and Shetty found that the Internet is increasingly becoming a primary source of health information, particularly among younger, more educated populations [16]. This shift presents both opportunities and challenges, as the quality of online health information can vary widely, necessitating efforts to ensure that accurate and reliable information is readily accessible. This preference for digital platforms suggests that future educational interventions should consider leveraging online resources and social media to disseminate information effectively.

The majority of parents (87.53%) reported having attended health education programs about newborn care, with a substantial portion of these programs including information on neonatal jaundice (88.43%). This suggests that while health education programs are widely attended and include relevant content, there is still room to enhance and tailor these programs to address specific areas of interest and concern more comprehensively. This positive trend highlights the importance of integrating neonatal jaundice education into broader maternal and child health programs. However, the 11.57% of parents who attended programs that did not cover jaundice points to a gap in the content of these programs, which should be addressed to ensure comprehensive newborn care education. The findings of this study align with those of a previous study conducted in Nigeria. Ogunlesi found that while there was some awareness of neonatal jaundice among Nigerian parents, knowledge gaps remained significant, particularly in rural areas [13]. These findings highlight the effectiveness of current information channels and the importance of expanding educational efforts, particularly through digital platforms, to meet the ongoing demand for knowledge about neonatal jaundice. This approach will help ensure that parents are well-informed and prepared to manage their newborn’s health effectively.

The study further revealed that several sociodemographic factors significantly impacted parental knowledge of neonatal jaundice, including age, gender, and education level. The study's findings indicated a significant association between age and knowledge of neonatal jaundice (p = 0.000). Parents aged 26-35 years had the highest awareness (153, 61.69%), followed by those aged 36-45 years (65, 65.66%). In contrast, only six (16.67%) parents aged 18-25 years had heard about neonatal jaundice. This suggests that older parents may have greater exposure to information or more experience with childcare, leading to better awareness of neonatal jaundice. These findings underline the importance of targeted educational interventions for younger parents, who may be less informed about neonatal health risks.

The analysis of parental awareness about neonatal jaundice reveals significant associations with several demographic factors. Awareness was notably higher among parents aged 26-35 years (61.69%) and 36-45 years (65.66%), while very few parents aged 18-25 years (16.67%) were aware of the condition. This age-related disparity, with a p-value of 0.000, suggests that older parents are more informed, possibly due to increased life experience or greater exposure to health information. Gender also played a crucial role, with females showing a significantly higher level of awareness (68.30%) compared to males (21.52%), as indicated by a p-value of 0.001. This discrepancy might reflect traditional gender roles in caregiving and health management, highlighting the need for targeted educational interventions to address gaps in male awareness. Previous studies have corroborated this gender difference in health knowledge, highlighting that women, particularly mothers, are more likely to seek health information and engage in preventive health behaviors than men [17]. This highlights a widespread need for more inclusive health education programs that involve both parents, especially in patriarchal societies where men may make crucial health decisions. Consequently, health education programs should consider incorporating strategies to actively involve fathers in neonatal care education to bridge this knowledge gap.

In contrast, marital status did not show a significant effect on awareness (p-value = 0.165), indicating that being single, married, or divorced/widowed does not substantially influence knowledge about neonatal jaundice.

However, educational level showed a significant association (p-value = 0.002), with higher awareness among those with secondary (66.21%) and tertiary education (75.28%), compared to those with no formal education (16.00%) or primary education (19.23%). This finding aligns with numerous studies that have consistently shown that higher educational attainment is associated with better health literacy and awareness [18]. This suggests that increased educational attainment correlates with better awareness, emphasizing the importance of educational outreach. Education equips individuals with the cognitive skills necessary to understand health information and make informed decisions regarding healthcare. Therefore, educational interventions aimed at improving knowledge of neonatal jaundice should particularly target parents with lower educational backgrounds. These findings reinforce the need for targeted education strategies to improve awareness among less informed demographic groups.

Occupation did not significantly impact awareness levels (p-value = 0.633), as awareness was relatively uniform across different employment statuses, including self-employed, private sector employees, and public sector employees. This indicates that factors other than occupation are more influential in determining awareness. Overall, these findings underscore the need for targeted educational programs that address specific demographic groups, particularly younger parents, males, and those with lower levels of formal education. Such tailored interventions could improve overall awareness and management of neonatal jaundice, leading to better health outcomes for newborns.

Recommendations

Enhancing parental knowledge and attitudes about neonatal jaundice is crucial for early detection, effective management, and prevention of complications like kernicterus. Based on the findings of this study, the following recommendations can be made for improving parental awareness and understanding.

Develop Clear, Culturally Appropriate Educational Materials

Use digital resources such as videos, animations, and online resources accessible through websites or social media platforms that can be shared with parents before and after delivery. Additionally, educational pamphlets, visual aids, and brochures, which are easy-to-understand materials in multiple languages that explain what neonatal jaundice is, its causes, signs, and symptoms, and when to seek medical help, can be distributed.

Integrate Education Into Routine Prenatal and Postnatal Care

Prenatal counseling, which includes discussions about neonatal jaundice including symptoms and complications as part of routine prenatal visits, especially for high-risk groups (e.g., families with a history of jaundice, preterm babies, and Rh-negative mothers), as well as postnatal classes incorporating neonatal jaundice education into childbirth classes and postnatal check-ups, can be implemented, including health education on the importance of early and frequent feeding. Midwives, nurses, and pediatricians can reinforce this information during routine visits to encourage discussions where parents can ask questions, express concerns, and get advice about neonatal jaundice management.

Empower Parents With Practical Monitoring Skills

Train parents on visual inspection on how to observe and identify early signs of jaundice (yellowing of the skin, eyes, etc.) and the areas to monitor (face, chest, and abdomen), as well as the significance of bilirubin levels and how routine screening is conducted, reassuring them about the safety and necessity of monitoring. Also, introducing mobile apps or telehealth platforms that guide parents in self-monitoring and connecting with healthcare providers if jaundice is suspected will be helpful.

Collaborate With Community and Support Groups

Establish support groups where experienced parents share their knowledge and experiences regarding neonatal jaundice, offering peer support to new parents through parent support networks. Community outreach programs, especially targeting underserved areas, to raise awareness about neonatal jaundice and its management, can be undertaken regularly to address cultural beliefs while promoting the benefits of early medical intervention.

Policymaking and Training of Healthcare Providers

Policymakers should consider integrating neonatal jaundice education into broader maternal and child health programs. Training healthcare providers to educate parents during antenatal and postnatal visits could also be instrumental in improving awareness and outcomes. Given the significant influence of education on health knowledge, efforts to increase literacy rates and access to education in the region could have long-term benefits for neonatal health.

Limitations of the study

As with all cross-sectional studies, this design limits our ability to establish causality. Relying on self-reported information introduces the risk of recall bias, where participants may inaccurately remember or report their experiences with neonatal jaundice, and the potential for socially desirable responses. Additionally, the list of causes of jaundice was not exhaustive, which suggests a need for greater inclusivity in future research. The knowledge and attitudes of parents regarding neonatal jaundice can rapidly evolve due to ongoing research, public health interventions, and shifts in population behavior. Since a cross-sectional study captures data from only one point in time, it may not reflect ongoing trends or fluctuations. To improve generalizability, future studies should consider increasing the sample size to enhance the power of the analysis [19].

## Conclusions

This study highlights significant gaps in parental knowledge of neonatal jaundice in Abia State, Nigeria, influenced by factors such as age, gender, and education level. These findings call for targeted educational interventions to raise awareness among less informed groups, particularly younger parents, men, and those with lower educational attainment. By addressing these gaps, it is possible to improve early detection and management of neonatal jaundice, ultimately reducing the risk of severe complications and improving neonatal health outcomes in the region.
